# Service quality and accessibility of healthcare facilities: digital healthcare potential in Ho Chi Minh City

**DOI:** 10.1186/s12913-022-08758-w

**Published:** 2022-11-19

**Authors:** Khanh Hung Le, Thi Xuan Phuong La, Markku Tykkyläinen

**Affiliations:** 1 Faculty of Urban Studies (FUS), University of Social Sciences and Humanities, Vietnam National University Ho Chi Minh City (VNU-HCM), Room A309, 10 - 12 Dinh Tien Hoang Street, Ben Nghe Ward, District 1, Ho Chi Minh City, Vietnam; 2 HCMC Institute for Development Studies, 28 Le Quy Don Street, Vo Thi Sau Ward, District 3, Ho Chi Minh City, Vietnam; 3 Department of Geographical and Historical Studies, University of Eastern Finland, P.O. Box 111, FI-80101 Joensuu, Finland

**Keywords:** Quality, Healthcare facilities, Accessibility, Digital healthcare, Telehealth, Remote care, Health services planning, Urban planning, GIS, Geospatial

## Abstract

**Background:**

Effective delivery of health services requires adequate quality in healthcare facilities and easy accessibility to health services physically or virtually. The purpose of this study was to reveal how the quality of healthcare facilities varies across the different parts of Ho Chi Minh City and how well residents (*N* = 9 million) can reach healthcare facilities. By demarcating the deficiently served areas of low accessibility, the study shows where urban planning and digital healthcare could improve accessibility to health services and the quality of services efficiently.

**Methods:**

The analysis utilised geocoded information on hospitals, clinics, roads and population and the data of the quality scores of healthcare facilities. Quality scores were analysed by hot spot analysis and inverse distance weighting. Accessibility and formation of travel time-based service areas by travel time distances were calculated using road network, driving speed and population data.

**Results:**

The results unveiled a centripetal spatial pattern of healthcare facilities and a similar pattern in their quality. Outside the travel time of 30 min for hospitals and 15 min for clinics, the deficiently served areas have a population of 1.1 to 1.2 million. Based on the results and the evidence of digital healthcare, this paper highlights how to develop and plan spatially effective service provision. Especially, it gives grounds to discuss how cost-effective digital healthcare could be applied to improve the accessibility and quality of health services in an urban structure of extensively varying accessibility to health services.

**Conclusions:**

The results bring up the need and the means for improving the quality of health services and their cost-efficient availability by location optimisation, road improvements and implementing digital healthcare provided by hospitals and clinics in the city. At the same, this study provides a multidisciplinary approach for planning more equal and efficient health service provision geographically.

## Background

The quality and accessibility of healthcare facilities play a crucial role in preventing and mitigating health problems. Health services planning designs health service delivery and performance, whereas urban planning develops urban infrastructure to meet the needs of health service providers and residents [1]. From these planning viewpoints, this study focuses on the quality and accessibility of healthcare facilities and shows where the deficiently served areas are located in Ho Chi Minh City (HCMC), the largest city in Vietnam. Thereafter, we explore how urban planning and various digital healthcare services would be suitable to improve quality and accessibility to health services on the big city scale.

Quality in healthcare facilities is usually classified as technical and perceived quality categories. The former refers to the level of health infrastructure and compliance with the instructions on the professionally defined practices and protocols of care according to current care guidelines and the later experiences and perceptions given usually by patients. World Health Organization (WHO) and researchers elsewhere have developed numerous indicators to assess the level of infrastructure and performance of healthcare facilities [2–4]. These principles are also followed in Vietnam where Vietnamese quality assessment scores of hospitals and clinics describe the quality of healthcare facilities [5, 6].

Globally, accessibility to health care varies strongly geographically by regional structure, affected by transport and population density. Travel time can take many hours or even days to reach primary healthcare services in the rural areas of less developed countries [7] whereas accessibility is substantially better in urban areas [8]. Many studies have investigated how to smooth out differences in accessibility in regions and countries as solving locational problems [9, 10]. Less has been discussed the spatial potential of digital healthcare as a part of health services planning and urban planning to abolish geographical differences in the accessibility and quality of health services cost-efficiently. Achieving a better spatial balance may require utilising telehealth and remote care with the latest digital healthcare technologies. Our aim is to fill this research gap, bringing digital healthcare into the part of urban and health services planning practices based on the empirical analyses of quality and accessibility of healthcare facilities in HCMC. This contribution expands the contents of planning doctrines also theoretically by developing geospatial thinking and methods into planning.

## Aim of the study

The aims of this study, presented as three research questions, are as follows: How does the quality of healthcare facilities vary across the different parts of HCMC, how well can residents reach current healthcare facilities, and how could remote care using telehealth and related technologies reduce spatial discrepancies in health care? The results of the empirical research are discussed in order to improve the accessibility and quality of health services paying attention on cost-effectiveness, urban planning, digital healthcare and the digital skills of the population.

## Research data and methods

In HCMC, healthcare facilities consist of hospitals and clinics. They are run by the government and private sector. Hospitals normally provide secondary and tertiary medical services, while clinics supply primary care and some basic treatments and first aid. Some clinics provide supplementary health services to hospitals.

The actors and stakeholders of HCMC health services planning are the government, the provincial level city government, private sector developers and subsidised social and NGO organisations and they execute the health services planning in the city [11]. HCMC Department of Planning and Architecture zones land for healthcare purposes and provides advice to solve the locational problems of healthcare.

We pay particular attention to how healthcare evolves in the expanding city and how services work in its periphery. The urban area of HCMC is classified into three major areas, named the established urban area, the new developing urban area and the suburban area. The three urban area classes mentioned above are named as outlined in the report of the World Bank [12]. The classification of the major area types follows the boundaries of administrative districts.

The healthcare facilities in the city comprised 134 hospitals and 260 clinics in 2020 and their attribute data were retrieved from the Information Portal of the HCMC Department of Health [13]. The attribute data covers name, address, type of ownership (public or private), type of service (general or specialised), scale of care (national, provincial or district level) and quality assessment score. All these data were converted into ArcGIS 10.6 software to use for the analysis stage.

This study applies public databases and GIS data for analysing the spatial health service system based on the address, road and population data and the quality data of healthcare facilities in HCMC. Based on the address information, the geographic coordinates of each individual healthcare facility were retrieved from Google Earth to the data set.

### Quality of healthcare facilities — hot spot analysis

The quality assessment scores, which describe the capability of providing services and estimate the quality of services, were derived from the quality assessment study implemented between June and December in 2019 [14, 15]. The standard set of quality criteria for hospitals were issued by the Vietnam Ministry of Health Portal [5] and the respective criteria for clinics by the HCMC Department of Health [6]. The quality scores of medical examination and treatment by facility are presented on a scale of one to five points and the results are available to citizens to know and choose when they need to use health services.

To unveil both high- and low-quality areas of healthcare facilities spatially in the city, the Getis-Ord ${G}_i^{\ast }$ local statistic (*z*-score) was applied [16]. The value is given by the Eq. (1):

1 $${G}_i^{\ast }=\frac{\sum_{j=1}^n{w}_{i,j}{x}_j-\overline{X}\ \sum_{j=1}^n{w}_{i,j}}{\textrm{S}\sqrt{\frac{n\ \sum_{j=1}^n{w}_{i,j}^2-{\left(\sum_{j=1}^n{w}_{i,j}\right)}^2}{n-1}}}$$

where *x*_*j*_ is the quality score by healthcare facility in location *j*;

*w*_*i*, *j*_is the distance between locations *i* and *j*;

*n* is the total number of locations;

$$\overline{X}=\frac{\sum_{j=1}^n{x}_j}{n};S=\sqrt{\frac{\sum_{j=1}^n{x}_j^2}{n}-{\left(\overline{X}\right)}^2}$$

The resultant *z-*scores and *p*-values with their coordinates indicate where the facilities of the high values and low values of quality are located in the city. This indicator works by showing the quality of a healthcare facility in the relationship with its neighbouring ones.

Having a statistically significant high *z*-score, the site is considered as a significant hot spot, indicating a high-quality healthcare facility. By contrast, a low negative z-score and a significant *p*-value indicate a significant cold spot, representing a poor level of quality. The higher or lower the *z*-score is, the larger is the deviation of hot spots and cold spots from the average. A *z*-score near zero indicates moderate quality of healthcare facilities. The inverse distance weighting (IDW) surface over the city was generated to show the smoothed geospatial variation of the quality of healthcare facilities across the city.

### Accessibility — service area analysis

The development of geocoded road and address databases, advanced population statistics and access to health records made possible to estimate healthcare accessibility using advanced GIS methods. There are two major ways to investigate healthcare accessibility; from the point view of the location of demand or combining demand with the supply of health services [17]. The former approach measures distance to the closest healthcare facility. The latter approach gives a broader view of the healthcare market by additionally including the regional availability of physicians [18] and it has also applied to improvement of a healthcare system [19].

This study finds out how residents reach current healthcare facilities and which areas should be targeted by virtual services to improve the provision of health services. Thus, we measure physical accessibility and, in addition to that, we consider the provision of telehealth and digital healthcare as recommended alternatives for areas of poor physical accessibility.

We estimated the travel distances of patients to the closest healthcare facility based on road data using ArcGIS Network Analyst. As the average speed of traffic was known, the temporal accessibility of a healthcare facility within given time limits was calculated. The service area of each healthcare facility was determined based on three maximum travel time durations by both hospitals and clinics. The layers of travel times by road were intersected with the HCMC administration layer at the smallest subdivision level consisting of 322 communes. The resulting intersected layers were used to work out how many residents live in the separate service area of each healthcare facility by means of the population density values of communes. After that, the total populations of the service areas based on three designated maximum travel times were summarised to the commune and district levels. In doing so, this study used the following preceding layers for data processing: (1) HCMC road network as polylines, (2) HCMC subdivisions as polygons, and (3) hospital and clinic locations as points.

The road and traffic data of HCMC is not detailed enough for a very accurate analysis, such as including speed limits and road conditions. Therefore, it is assumed that the driving time to and from healthcare facilities complies with the average driving speed in the city. There is no universally recommended travel time limit for the maximum travel time of patients to a healthcare facility [20]. It is supposed that the maximum one-way driving time in each service area is 30 min for hospitals and 15 min for clinics. Clinics provide a narrower range of services than hospitals. Thus, the customer selects the health service between two types of service according to need. Motorbikes are the most frequent vehicles in everyday traffic in HCMC [21]. The average trip length by motorbike is 8.17 km and by car 10 km and the average speed is about 20 km/h for both vehicle modes in HCMC [21]. At that speed, 10 km is driven within 30 min. Three different travel time limits were set for both hospitals and clinics in the service area analysis: 10, 20 and 30 min for hospitals and 5, 10 and 15 min for clinics. The travel time zones define concentric service areas and their area and population coverages for hospitals and clinics.

## Results

### Spatial distribution patterns of healthcare facilities

**Fig. 1:**
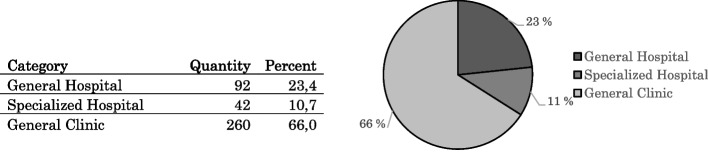
Healthcare facilities by the type of service in HCMC in 2020

In HCMC, 394 healthcare facilities performed medical examinations and treatment in 92 general hospitals, 42 specialised hospitals and 260 clinics (Fig. 1). The healthcare facilities are divided administratively into three hierarchical levels: national, provincial and district. Eight healthcare facilities are managed and operated at the national level, 41 at the provincial level and 345 at the district level.

**Fig. 2:**
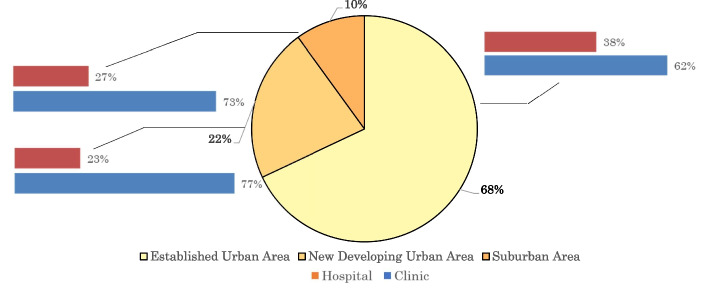
Distribution of hospitals and clinics by area type in HCMC in 2020

Sixty-eight percent of healthcare facilities are located in the established urban area (Fig. 2). Hospitals make up the largest share (38%) of healthcare facilities there whereas clinics have the largest share (77%) in the new developing urban area. Although clinics have the larger shares of the healthcare facilities both in the new developing urban area and the suburban area than in the established urban area, each clinic in the new developing area must serve almost the double number of patients and in the suburban area almost the triple number of patients compared with a clinic the established urban area. So far, there has been no tendency to relocate hospitals or clinics from the densely populated established urban area to the new developing urban area or the suburban area.

**Fig. 3:**
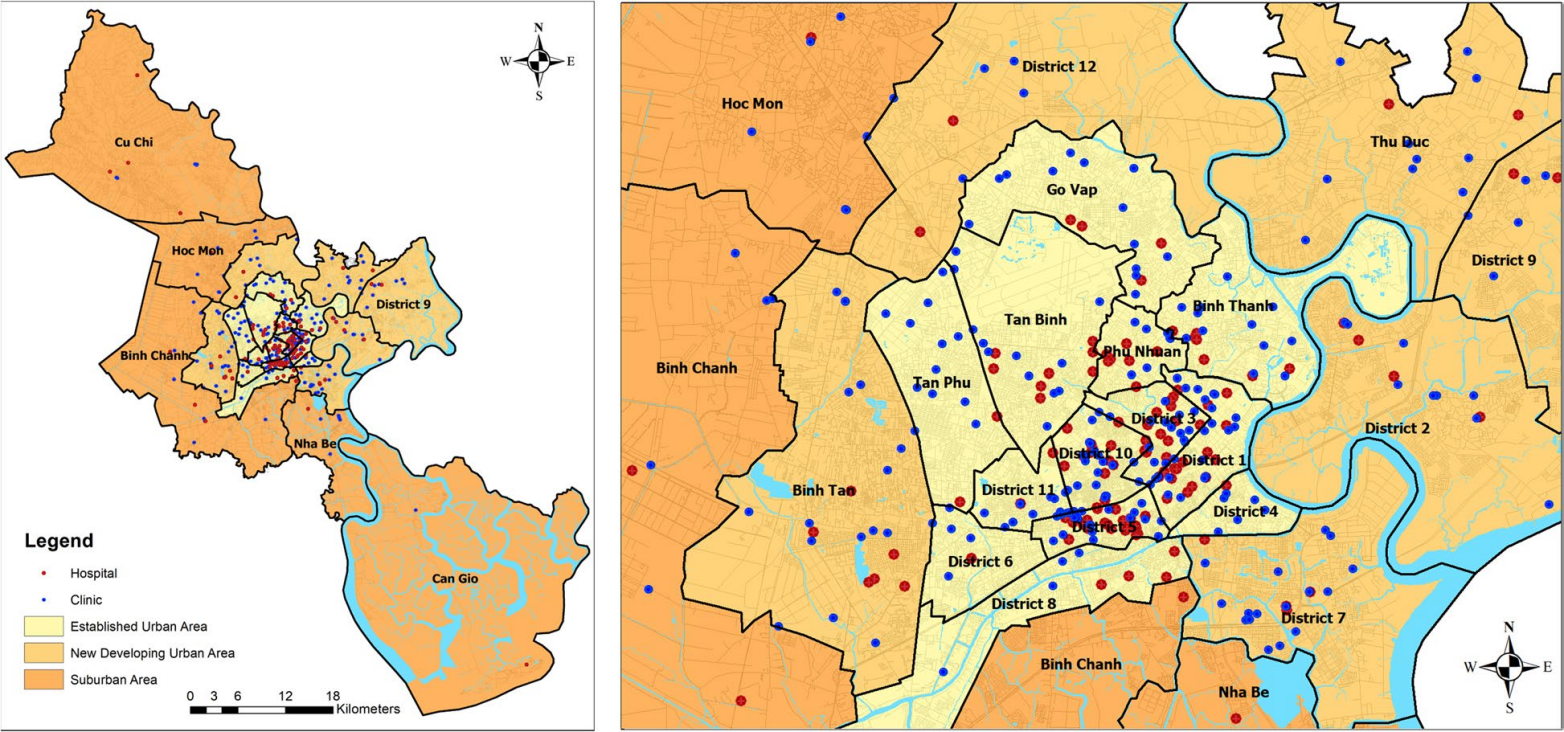
Distribution of hospitals and clinics in HCMC in 2020

The established urban area, Districts 1, 3, 4, 5, 6, 8, 10, 11, Binh Thanh, Phu Nhuan, Go Vap, Tan Binh and Tan Phu (Fig. 3), had 15,188 residents per healthcare facility in 2020. The ratio indicates high supply, which also reflects the concentration of health services and especially national level specialised care in the established urban area. District 1 and a part of District 3 constitute the CBD area of the city and hospitals are concentrated on the high-density population circle, named as zone A [22]. Zone A includes the established urban area except Districts 6 and 8.

The new developing urban area consists of Districts 2, 7, 9, 12, Thu Duc and Binh Tan, and it had 33,729 residents per healthcare facility whereas the suburban area, comprising Hoc Mon, Cu Chi, Nha Be, Binh Chanh and Can Gio, had 50,978 residents per healthcare facility. Thus, the new developing urban area has attracted healthcare facilities much better than the suburban area. As the population density is high and streets are congested in the inner city, it is expected that, as the city expands in the new developing urban areas, new healthcare facilities will be set up there, attracted by rising demand.

### Hot and cold spots of quality of healthcare facilities

Hospitals and clinics have separate quality criteria. In 2019, HCMC had 330 healthcare facilities undergoing quality assessment. Hospitals were assessed for their service quality according to five main criteria: (i) patient-centred service (19 criteria, such as directing, welcoming, guiding and giving first aid to patients, the conditions of facilities to serve patients), (ii) human resource development and management (14 criteria), (iii) professional competence and skills (35 criteria), (iv) quality improvement activities (11 criteria), and (v) particular criteria for specialisation (four criteria).

**Fig. 4:**
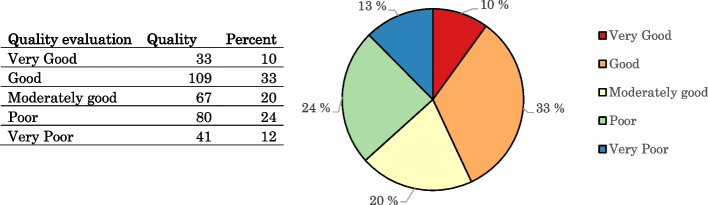
Results of quality assessment of healthcare facilities in 2019

Clinics were assessed according to 23 criteria, including factors such as human resources, medical equipment, examination and treatment processes, IT applications, incidents and their analysis system, services for patients, price transparency, infection control and prevention measures, and medical waste management. The rating scale of both hospitals and clinics is divided into five levels, with scores from 1 to 5 for each criterion. A score of less than 2 means that the service quality of a healthcare facility is very poor, from 2 to less than 2.5 poor, from 2.5 to less than 3 moderately good, from 3 to less than 4 good and from 4 to 5 very good (Fig. 4).

The majority of healthcare facilities reached at least a moderately good level and 43% of facilities were rated at least as good or very good (Fig. 4). Healthcare facilities at the very good level accounted for 10%. All hospitals managed at the national level requirements were rated as very good. Only 12% of healthcare facilities were rated as very poor in quality; they were mainly clinics.

**Fig. 5:**
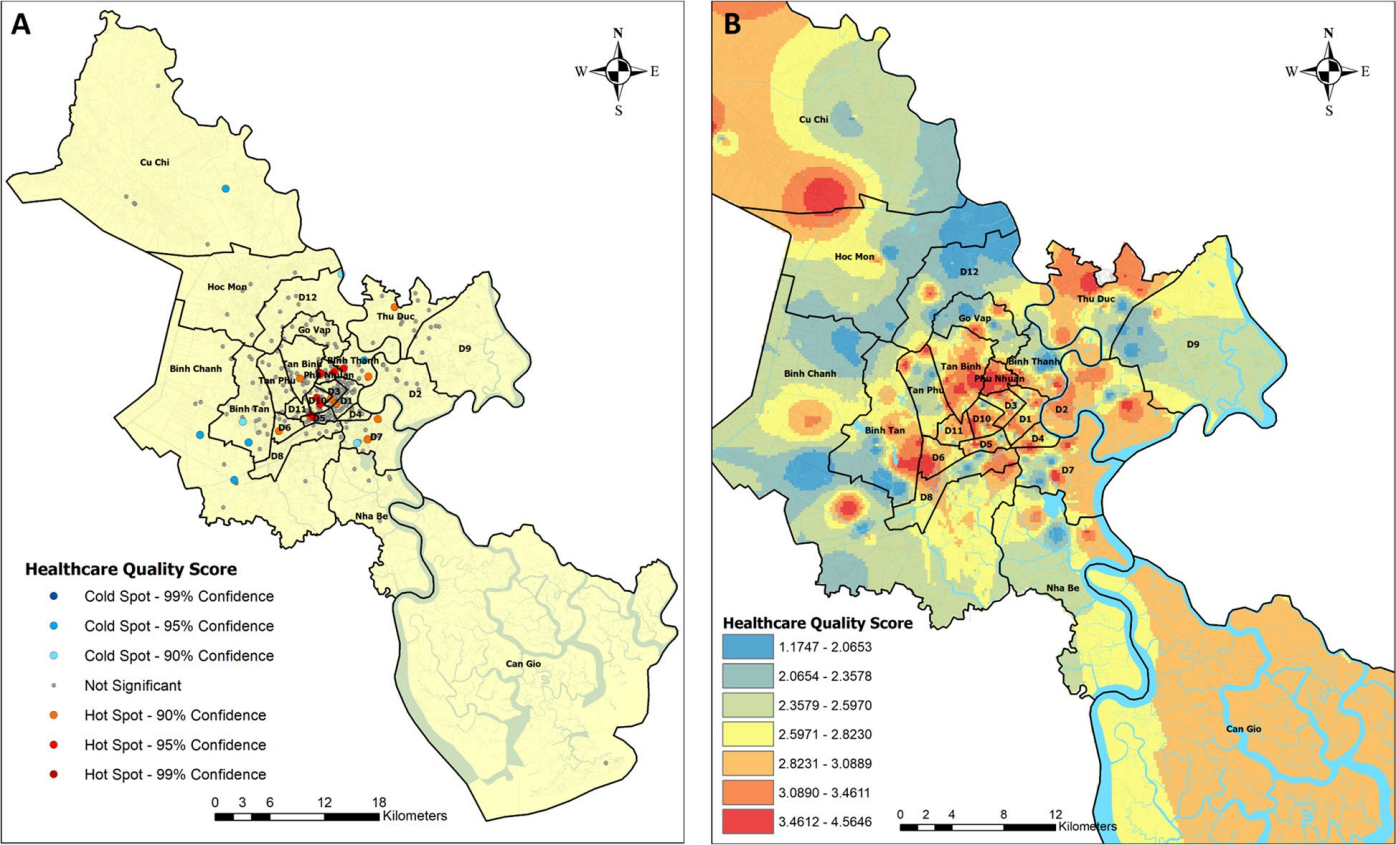
Hot and cold spots of the quality of healthcare facilities and the spatial reach of quality in 2019. **A** on the left: Hot and cold spots of quality. **B** on the right: IDW quality surface

Hot spot analysis reveals where the quality of a healthcare facility is statistically significantly high or low at the 90, 95 and 99% confidence intervals. The darker the colour of a dot, the tighter the criterion (Fig. 5A). Red dots on the map as hot spots unveil where the best healthcare facilities are located, whereas blue dots as cold spots unveil where the poorest healthcare facilities are located and where improvements in quality are necessary. Grey dots indicate that the quality of healthcare facility is ranked moderate. The quality of medical examination and treatment varies from poor to good in the grey healthcare facilities.

The significant hot spots of high quality are located in the established urban area, as in District 1, District 5, District 10 and Phu Nhuan District, at the confidence intervals of 90% and over. In this group of districts, most of the hot spots represent national-level hospitals, such as University Medical Center, Cho Ray Hospital, and provincial-level hospitals, such as The People’s 115 Hospital, People’s Gia Dinh Hospital, Hung Vuong Hospital, Binh Dan Hospital and National Institute of Medicine and Pharmacy. These hospitals are upper-level and established large-scale public general hospitals. They are well-equipped and offer high-quality special medical care. The only hot spot district-level general hospital is Thu Duc District Hospital in the northeast. Both Tu Du District Hospital and national-level Children’s Hospital No.1 have a good reputation for paediatric and reproductive health services [23, 24]. Both belong to the hot spot group of hospitals.

Some hot spots are in the recently constructed high-end residential areas, such as Vinmec International Hospital in Vinhome Central Park in Binh Thanh District in the established urban area and Tam Duc Heart Hospital in Phu My Hung in District 7 in the new developing urban area. High-quality clinics appear in District 7, such as the General Clinic of Branch No.1 of Phuc An Hospital System Corporation and the General Clinic of Tam Duc Heart Hospital. Their locational choices indicate that high-quality private healthcare facilities tend to follow emerging service demand in the new developing urban area. Usually, healthcare facility construction is boosted by tax incentives. These facilities comprise social infrastructure to supply professional health services to meet the demand for high health standards in middle-class neighbourhoods. In contrast, cold spots indicate low quality healthcare facilities located in District 9, District 12 and Binh Tan District in the new developing urban area. Cold spots exist also in Binh Chanh District and Cu Chi District in the suburban area (Fig. 5A). Healthcare facilities need attention in these districts.

The IDW interpolation surface of quality scores was estimated the spatial reach of the quality of healthcare facilities. Its influence areas are shown by the seven classes of the quality score values (Fig. 5B). Residents in the established urban area have a very good chance of getting health services at the moderately good, good or very good levels. Healthcare operators have extended the service provision of high quality in the recently constructed large high-end residential areas. Nevertheless, many healthcare facilities provide services at the very poor or poor level in some parts of both the new developing urban area and the suburban area, as depicted in blue areas in the fringe areas of outer districts (Fig. 5B). However, in north-western HCMC, Cu Chi General Area Hospital had a good level of quality; it operates where the density of facilities is low and clinics got relatively low scores. In Can Gio in the south, the quality is moderately good. The IDW surface concretises the fact that most high-quality hospitals are located in the central and most populous areas, which creates potential to provide high-quality services with high population coverage, while the new developing urban and suburban areas have district level hospitals and general clinics on a smaller scale, supplying health services to a geographically vast area; these are often lower quality facilities.

### Travel time-based service areas of healthcare facilities

**Fig. 6:**
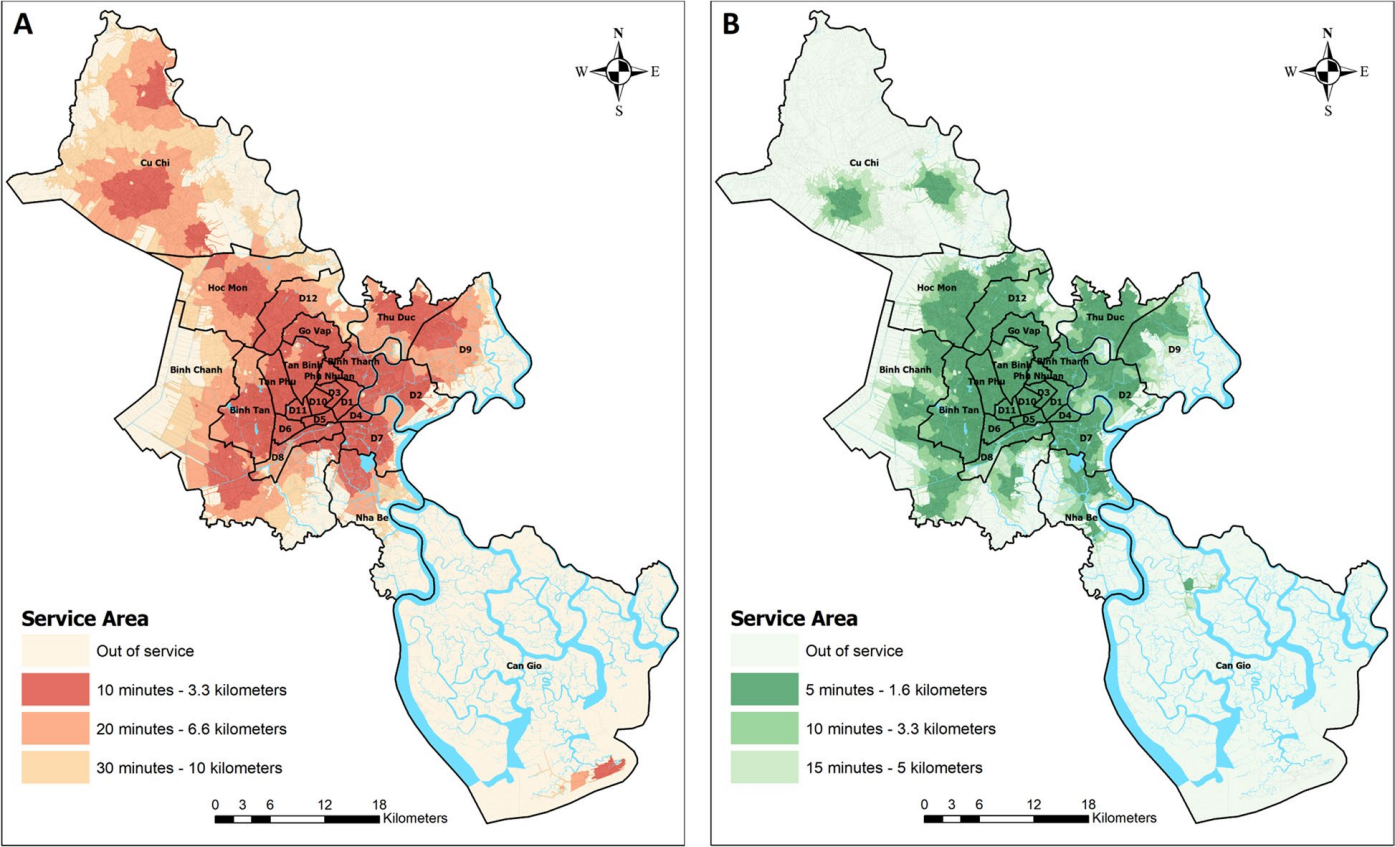
Travel time-based service areas of hospitals and clinics in 2020. **A** on the left: hospitals, **B** on the right: clinics

Accessibility was estimated separately for hospitals and clinics to unveil well-served and deficiently served areas. Three travel time zones were set for both hospitals and clinics to create service areas based on travel times at the average speed of 20 km/h. The inner and smallest zone constitutes the maximum of 10 min travel time to a hospital and 5 min to a clinic. The middle zone means a travel time from above 10 min to 20 min to a hospital and from above 5 min to 10 min a clinic, and the outer zone represents travel times from above 20 min to 30 min to a hospital and from above 10 min to 15 min to a clinic. The spatial pattern of accessibility manifests itself the circular surfaces of travel time zones around each facility and the service areas of both hospitals and clinics are made up of these travel time accessibility zones (Fig. 6).

The travel time-based service areas of hospitals within the time limits of 10 min covered all residents in District 4 (Table 1, Fig. 6A). In the entire established urban area, 10-min travel time areas accounted for 88% of the population and 94% of the land. Hospital density and road and alley networks are high in this area which makes urban residents’ travels to healthcare facilities easy.

**Table 1:** Temporal accessibility of healthcare facilities by district in HCMC

District	Area (km^**2**^)	Population 2019	Hospitals						Clinics					
			Area Coverage (%)			Population Coverage (%)			Area Coverage (%)			Population Coverage (%)		
			10 min	20 min	30 min	10 min	20 min	30 min	5 min	10 min	15 min	5 min	10 min	15 min
**ESTABLISHED URBAN AREA**														
**Total**	**141.96**	**4,070,480**	**93.82**	**99.26**	**99.67**	**87.86**	**97.94**	**99.11**	**86.63**	**93.17**	**98.56**	**96.47**	**98.62**	**99.67**
District 1	7.75	142,625	99.81	99.92	99.96	99.77	99.96	99.98	99.75	99.96	99.98	99.82	99.91	99.96
District 3	4.93	190,375	99.91	99.95	99.99	99.75	99.86	99.97	100	100	100	100	100	100
District 4	4.18	175,329	100	100	100	100	100	100	99.82	99.91	99.98	99.88	99.93	99.99
District 5	4.28	159,073	99.89	99.94	99.98	99.92	99.96	99.98	99.93	99.96	99.98	99.91	99.94	99.98
District 6	7.16	233,561	99.40	99.54	99.65	99.33	99.48	99.61	98.84	99.47	99.60	99.02	99.52	99.64
District 8	19.07	424,667	77.87	97.01	98.59	57.02	92.52	96.44	62.48	75.94	95.56	82.11	91.73	98.33
District 10	5.72	234,819	99.75	99.85	99.93	99.87	99.92	99.97	99.88	99.92	99.96	99.78	99.84	99.93
District 11	5.13	209,867	100	100	100	100	100	100	100	100	100	100	100	100
Binh Thanh	20.69	499,164	91.81	98.84	99.52	69.03	92.15	96.75	75.66	82.90	95.24	96.08	97.60	99.33
Go Vap	19.69	676,899	96.04	99.75	99.91	94.64	99.62	99.85	98.92	99.56	99.85	99.18	99.69	99.90
Phu Nhuan	4.91	163,961	100	100	100	100	100	100	100	100	100	100	100	100
Tan Binh	22.46	474,792	96.62	99.53	99.83	87.87	98.45	99.50	71.44	93.77	99.41	91.98	98.27	99.81
Tan Phu	16.01	485,348	92.28	99.81	99.91	87.61	99.78	99.91	99.70	99.80	99.92	99.73	99.82	99.92
**NEW DEVELOPING URBAN AREA**														
**Total**	**352.14**	**2,934,441**	**55.66**	**85.61**	**88.72**	**48.83**	**81.09**	**86.14**	**50.17**	**64.90**	**72.40**	**74.22**	**86.22**	**90.17**
District 2	49.92	180,275	67.77	79.86	81.54	56.04	74.97	76.95	45.37	70.06	76.91	59.62	78.20	81.51
District 7	35.27	360,155	74.43	88.06	89.19	58.49	80.17	81.83	64.54	79.44	81.72	78.58	87.64	89.12
District 9	114.29	397,006	38.78	80.39	83.75	15.85	46.73	53.74	20.31	28.53	39.07	50.60	63.22	70.87
District 12	52.76	620,146	61.51	86.94	93.51	42.03	78.17	90.18	57.98	77.61	91.19	74.43	87.22	94.02
Binh Tan	51.99	784,173	68.21	98.34	99.44	64.52	98.33	99.49	86.50	98.95	99.52	89.69	98.85	99.47
Thu Duc	47.90	592,686	49.43	86.94	90.79	49.21	86.77	91.34	67.78	84.62	90.23	71.16	85.42	90.05
**SUBURBAN AREA**														
**Total**	**1615.66**	**1,988,161**	**12.23**	**28.72**	**39.94**	**16.11**	**45.23**	**65.38**	**7.96**	**13.67**	**19.39**	**31.16**	**46.12**	**56.28**
Binh Chanh	252.83	705,508	18.51	49.02	71.95	13.27	39.27	61.41	20.49	39.07	51.86	27.31	52.40	66.03
Can Gio	718.58	71,526	2.14	2.74	3.22	0.59	1.08	1.44	0.18	0.28	0.81	0.23	0.36	1.15
Cu Chi	434.64	462,047	15.73	42.53	63.19	11.97	41.44	65.32	4.33	8.77	16.69	8.86	15.44	26.01
Hoc Mon	109.12	542,243	41.35	80.98	93.56	26.30	67.96	88.43	36.74	51.16	62.30	58.99	70.94	77.46
Nha Be	100.50	206,837	21.80	46.99	63.18	13.74	29.69	40.76	16.56	25.97	35.68	31.91	43.96	54.14

In the new developing urban area, within the 10-min service radius of hospitals, the service areas covered 49% of the population and a half of the land, whereas in the suburban area, the coverage was just over 16% and one eighth, respectively (Table 1). Within the service radius of 20 min, the service areas of hospitals were widened to cover 82% of the population of the new developing urban area, but to less than a half of the population of the suburban area (Table 1).

The maximum travel time of 30 min increased the covered population to nearly 86% in the new developing urban area. Hence, only about every tenth person does not reach a hospital within 30 min. In the suburban area, within the 30-min service radius of hospitals, the share of included people was 65% (Table 1).

Residents who were outside the 30-min service areas of hospitals total up to 1,131,261 in the city. Of those outsiders, 3% resided in the established urban area, 36% in the new developing urban area and 61% in the suburban area. The 30-min service areas of 134 hospitals cover 1099 km^2^, which comprises 52% of the HCMC municipal area. However, 7,861,821 residents, representing over 87% of the population, reached a hospital within 30 min.

Two sub-district areas have fallen behind in the new developing urban area; some residential areas in District 9 and District 2 have a poor accessibility to a hospital. This is caused by the blocking impact of Ring Road 2 from Phu My Bridge to Sai Gon High-Tech Park and the CT.01 expressway from Long Thanh towards Dau Giay. The construction of the ring road and the expressway was intended to improve traffic circulation and the transport of commodities through the city. However, this traffic arrangement pulled down functional areas and increased distances locally and hence travel times to health facilities. The consequent increase in travel times rolls back accessibility to hospitals in Districts 9 and 2 (Table 1). A reconciliation of transport and urban planning and health services planning would have been better.

Clinics are composed of smaller time-based service areas than hospitals (Fig. 6B). They are numerous in the established urban area, where the service areas of clinics were large enough for covering 96% of the population within the maximum of 5 min travel time to a clinic. Coverage increased to 100% within the longest travel time boundary of 15 min (Table 1).

In the new developing urban area, 74% of the population reached the nearest clinic within 5 min, 86% of the population within 10 min and 90% of the population within 15 min (Table 1). In the suburban area, a clinic was reached within 5 min by 31% of the population, within 10 min by 46% of the population and within 15 min by 56% of the population (Table 1). Suburban residents reach clinics best in local agglomerations where settlement is predominantly concentrated.

The total service area of 260 clinics covers 708.2 km^2^, which comprises 34% of the HCMC municipal area. Clinics were reached within 15 min by 7,821,818 people in the city, equating to nearly 87% of the city population. Thus, the great majority of residents can reach a clinic within a quarter of an hour. Because service areas are formed using the shorter maximum travel time to clinics than to hospitals, it leads to the smaller total service area of clinics than the total service area of hospitals, but the number of people potentially served is about the same as in the service areas of hospitals. The number of clinics is almost twice of that of hospitals and the higher density of clinics makes possible to a large population to reach the nearest clinic within very short travel times.

Clinics are spatially concentrated in the most urbanised core area (Fig. 3). In addition to a high demand due to high population density, a reason for the spatial concentration is that some clinics operate by providing intermediate and supporting functions such as offering diagnostic, therapeutic and outpatient services for hospitals and patients who have no serious health problems and do not require a bed or to be admitted for overnight care. Many such clinics are privately owned and market-oriented, as are many of their customer hospitals. Clinics located in the suburban area supply mostly primary care and some basic treatments and first aid. Both clinics and hospitals share similar characteristics in their spatial distribution and accessibility for the benefit of central areas.

## Discussion

### Interpretation of results

A few limitations need to be considered in interpreting the results. Public transportation is based on buses and is limited in HCMC. Hence, bus transports cannot have significant impact on travel time estimates. The average speed observed in the city was used because the GIS road data of the city was not detailed enough to depict differences in road quality and speed. Therefore, these calculations give a rough estimate of accessibility to healthcare facilities. If observed speeds by each road would have been available, they would probably have extended some service areas in the new developing and suburban areas. Because the address data of residents was not available, the population density data of 322 communes, whose median size is 1.1 km^2^, were used to determine the location of residents. Very likely, this estimation of distances is reliable enough at the level we interpreted the results.

Broadband accessibility describes the performance of telecommunication which is an important factor in providing digital healthcare successfully [25]. We had no such data available on HCMC. Similarly, there were no age and employment data of the population at the small-area or grid level. That data would help healthcare research and planning and urban planning as they allow spatial socio-economic conditions to be included in accessibility analysis.

Since hospitals and the high quality of facilities are concentrated in the established urban area, a part of patients must travel farther than their nearest hospital or clinic to treat special health problems. Demand for special health care increases visits to healthcare facilities located mostly in the established urban area, but so far there is no suitable data to estimate the use of special health care services.

Both the quality and accessibility of healthcare facilities are in HCMC at their best in the established urban area, as is usual in megacities [8, 26]. Especially in the new developing urban area, healthcare facilities have been constructed along with real estate investments that mitigate shortages in supply for health services. Remote locations mean low market with sporadic demand, leading to poor quality and poor accessibility. The alleviation of this drawback calls for attention, measures and new solutions.

Travel times to healthcare facilities are short for the great majority of residents in HCMC. Short travel times are similar to obtained in Shenzhen [8], which is a rapidly growing megacity of about the same areal size, but slightly more populous. One reason for rapid accessibility is that self-driving by motorbike or car are the most popular modes of transport in HCMC. They are faster transport modes than sparse public transportation. Gu et al. [27] obtained similar results in Shanghai, where Zhang et al. [28] found that public transport-based accessibility is dependent on routes, leading to a varying pattern of accessibility. Similar findings have been obtained from Kaifeng [29].

The spatial organisation of both hospitals and clinics are structured centripetally in HCMC. The centripetal spatial pattern of health services is typical in urban areas [30]. It is caused by the distribution of the population; where the population is dense and solvent, the density of hospitals and clinics is high, as expected. Similarly, those factors are reasons for establishing hospitals and clinics in the new developing urban area. It improves accessibility, as HCMC is a more compact city than highly suburbanised automobile-oriented cities [31]. The demand for supplementary services increases the clustering of clinics to near hospitals. Clustering makes possible high specialisation and creates supporting health services via clinics. Moreover, clinics supply primary care especially outside the established urban area where hospitals are sparse.

### Prospects and recommendations — planning, digital health and digital skills

Although most residents can readily reach a health service facility, still about 13% of the city’s population cannot reach any hospital within 30 min or a clinic within 15 min at the average speed of traffic. Accessibility is poorest in the suburban area, where more than one third of the residents live beyond the maximum travel time limits of 30 min and 15 min. Road improvements would shorten travel times, but the impacts would be small. In future planning, district level hospitals and primary care clinics should be located more optimally because their accessibility plays an important role for residents. Supplemental planning can be effective. As an example, Reshadat et al. [9] applied optimisation in the GIS-based planning of new supplementary healthcare facilities in order to provide better healthcare coverage for the population of Kermanshah. Urban research and planning are hence of use in health services planning. Planning is very sector-based in Vietnam [12, 32]. We recommend strengthening the interplay between urban planning, health service planning, housing policy and research.

Telehealth provides an alternative to traditional in-person consultations. Such health services are valued as highly accessible, time-saving and contributing to ecological sustainability [33]. The COVID-19 pandemic multiplied the utilisation of telehealth consultations in Southern Ontario [34] and Brazil [35] showing that a rapid adoption of telehealth is possible. The diffusion of telehealth is dependent on digital skills and access to mobile and information technologies and communications networks. In Vietnam, there were 143 mobile cellular subscriptions per 100 people in 2020 [36] and 70% of the population used the Internet in 2020 [37]. Especially the density of mobile phones is high. A register-based study of the population of Stockholm found that the probability of telehealth consultation increased with younger age, higher education attainment and higher income [38]. According to a study of 403 adults in Mozambique, customers are willing to use telehealth in cases of mild illness, cheaper price and follow-up consultation [39]. Thus, the propensity to use telehealth services is dependent on the age, digital skills, incomes, affordability, disease and phases of care. Vietnam is technologically progressive and has a young population, but the impact of local socio-economic circumstances on the assimilation of telehealth services should also be considered in urban health services planning.

Campanella et al. [40] observed that digital healthcare not only partially lead to a higher level of customer satisfaction, but also to better organisational performance of public health companies. Such technology can also enhance care protocols and decrease the number of visits to healthcare facilities, as the part of visits can be replaced by self-monitoring and remote follow-up care [41] and using algorithms to select drugs that require fewer follow-up visits to laboratories from fringe areas [42, 43]. In pre- and postoperative telemedicine consultations services given by Korean physicians to Vietnamese patients, a robot-based system for telemedicine decreased in-person contact, travel and cost [44]. At its simplest, consultation and disease surveillance can be improved through teleconferencing and basic mobile technology [45–47]. Health applications in smartphones are seen as useful among youth and young adults in Vietnam [48]. According to the same study, 66.3% of respondents downloaded the mHealth applications for disease prevention. Hence, we recommend telehealth applications for acute and curative condition among youth and young adults who embrace them quickly. In the light of these experiences, telemedical services would improve the availability of health services in HCMC. It is expensive to reallocate the spatial distribution of healthcare facilities, so quick and efficient ways must be tailored to improve access to health services of adequate quality where necessary.

Digital healthcare development can be summarized as a planning framework that combines supply and demand. Both perspectives are needed to develop successful digital healthcare services. On the supply side, central factors are the quality and readiness of digital healthcare delivery, the rules of authorities, the state of urban infrastructure and planning, broadband accessibility and the decisions of stakeholders. On the demand side, central factors are patients’ willingness to use digital healthcare, their digital abilities and socio-economic factors, accessibility in various forms, and communication possibilities.

## Conclusions

Our findings show that the quality of healthcare facilities and accessibility deteriorate significantly with distance from the urban core. In addition to traditional measures of investments in roads and transport systems, time-based accessibility and the spatially uneven quality of health services can be improved by location optimisation and developing digital healthcare of uniform quality. As accessibility and quality in health care vary, there are reasonable grounds to develop digital healthcare with no delay in HCMC. Hospitals and clinics would be recommended to start to provide digital healthcare services for which they have very good readiness. Such developing healthcare facilities would also serve examples to others. The spatial development of digital health care should be monitored and guided by using quality assessment studies and to pay attention how especially the treatment by digital healthcare impacts in the deficiently served areas and individuals in general.

In line with the results and evidence from other studies, we recommend that health services planning promotes the deployment of digital healthcare, such as telehealth, self-monitoring, remote follow-up and health applications to improve the availability, quality and cost-effectiveness of health services in the city and in particular in the conditions of a thin healthcare market when sporadic demand for health services prevails. Moreover, the deployment of digital healthcare tools would bring health benefits by reducing the need for travel. The methodological contribution of this study is the linking of digital healthcare solutions to both urban planning and health services planning and showing how to determine the digital healthcare potential of deficiently served areas in a big city. Our approach provides procedures to assess and design a more equal virtual and physical healthcare system.

## Data Availability

All data is based on public databases referenced in the text and reference list. Quality material available: http://www.medinet.hochiminhcity.gov.vn/benh-vien/ket-qua-danh-gia-chat-luong-benh-vien-thuoc-nganh-y-te-tphcm-nam-2019-cmobile16308-22365.aspx and http://medinet.hochiminhcity.gov.vn/quan-ly-chat-luong-kham-chua-benh/ket-qua-danh-gia-chat-luong-phong-kham-da-khoa-tren-dia-ban-thanh-pho-nam-2019-c8-21632.aspx

## Abbreviations

GIS: Geographic Information Systems
HCMC: Ho Chi Minh City
IDW: Inverse Distance Weighting
WHO: World Health Organisation
